# Financial and Work-flow Benefits of Reducing Avoidable Hospitalizations of Nursing Home Residents

**DOI:** 10.1007/s12603-021-1650-2

**Published:** 2024-01-04

**Authors:** Marilyn Rantz, A. Vogelsmeier, L. Popejoy, K. Canada, C. Galambos, C. Crecelius, G.L. Alexander

**Affiliations:** 1 University of Missouri Sinclair School of Nursing, Columbia, USA; 2 University of Missouri, Columbia, USA; 3 University of Wisconsin-Milwaukee, Milwaukee, USA; 4 BJC HealthCare, St. Louis, USA; 5 Columbia University, New York, USA

**Keywords:** Nursing homes, avoidable hospitalizations, advanced practice registered nurses (APRNs), INTERACT, revenue, workflow in nursing homes

## Abstract

**Objectives:**

1) Explain the financial benefit of potential revenue recapture (PRR) for non-billable days due to hospitalizations of nursing home (NH) residents using a six-year longitudinal analysis of 11 of 16 NHs participating in the Missouri Quality Initiative (MOQI); and 2) Discuss the work-flow benefits of early detection of changes in health status using qualitative data from all MOQI homes.

**Design:**

A CMS funded demonstration project with full-time advanced practice registered nurses (APRN) and operations support team focused on reducing avoidable hospitalizations for long stay NH residents (2012–2020).

**Setting and Participants:**

Setting was a sample of 11 of 16 US NHs participating in the CMS project. The NHs ranged in size between 121 and 321 beds located in urban and rural areas in one midwestern geographic region.

**Methods:**

Financial and occupancy data were analyzed using descriptive methods. Data are readily available from most NH financial systems and include information about short and long stay residents to calculate non-billable days due to hospitalizations. Average hospital transfer rates per 1000 resident days were used. Qualitative data collected in MOQI informed the work-flow benefits analysis.

**Results:**

There was over $2.6 million in actual revenue recapture due to hospitalization of long stay residents in the 11 participating NHs during five years, 2015–2019, with 2014 as baseline; savings to payers was more than $31 million during those same years. The PRR for both short and long stay residents combined totaled $32.5 million for six years (2014–2019); for each NH this ranged from $590,000 to over $5 million. On average, an additional $500,000 of revenue each year per 200 beds could have been recaptured by further reducing hospitalizations. Workflow improved for nurses and nursing assistants using INTERACT and focusing on early detection of health changes.

**Conclusions:**

Reducing avoidable hospitalizations reduces costs to payers and increases revenue by substantially recapturing revenue lost each day of hospitalization.

**Implications:**

Focusing nursing staff on early illness recognition and management of condition changes within NHs has benefits for residents as the stress of hospital transfer and resulting functional decline is avoided. Nurses and nursing assistants benefit from workflow improvements by focusing on early illness detection, managing most condition changes within NHs. NHs benefit financially from increased revenue by reducing empty bed days.

**H**ospitalizations for nursing home (NH) residents are common; however, some researchers suggest that 60–67% of all hospitalizations from NHs are avoidable (1, 2). Avoidable hospitalizations are costly. One estimate suggests costs to Medicare (in 2006 dollars) of more than $2 billion for avoidable hospitalizations of NH residents (3); another estimates Medicare and Medicaid expended $2.6 billion (in 2005 dollars) for avoidable hospitalizations of dual-eligible NH residents (4). Moreover, in 2016, each hospitalization has higher costs per stay ($13,600) to Medicare compared to other payers ($9,300–$12,600) (5). Avoidable hospitalizations are costly and unsustainable given the economic strains in the United States.

When hospitalized, NH residents often experience physical, mental, and overall functional decline with unintended consequences that may contribute to permanent loss of function, decline, or death (6, 7, 8). Detecting changes in health conditions early, managing needed care within the familiar environment of the nursing home, not only reduces those consequences, but also speeds recovery and residents are more satisfied with the care from people they who know them and their preferences (9). Both the costs of avoidable hospitalizations and the negative health consequences for residents make a strong case for national efforts to develop ways to reduce avoidable hospitalizations for NH residents.

The Missouri Quality Initiative (MOQI) was one of seven sites selected to participate in the national Centers for Medicaid and Medicare Services (CMS) Innovations Center, Initiative to Reduce Avoidable Hospitalizations among Nursing Facility Residents (2012–2020). The goals of the CMS Initiative for long-stay NH residents were to reduce avoidable hospital admissions and readmissions, improve resident health outcomes, improve the process of transitioning between inpatient hospitals and NHs, and reduce overall healthcare spending without restricting access to care or choice of providers.

MOQI was designed to test ways to effectively reduce avoidable hospitalizations from NHs. Key components of the MOQI intervention include a full-time advanced practice registered nurse (APRN) in each facility, and an operations support team (9, 10) working across all participating facilities to provide support for the implementation of Interventions to Reduce Acute Care Transfers (INTERACT) processes (11), end of life care, care transitions, and health information technology (12, 13). MOQI was one of the best performing programs (14) with 40% reduction in all-cause hospitalizations and 58% reduction in potentially avoidable hospitalizations (p-value <0.001) (15).

While effectively reducing avoidable hospitalizations is of benefit to NH residents as well as financial benefit to Medicare and Medicaid, it is also important to consider potential benefits to NHs and staff who care for residents. The purpose of this article is two-fold: 1) to explain the financial benefit of potential revenue recapture (PRR) for non-billable days due to hospitalizations of NH residents (both long and short stay) using results of a six-year longitudinal analysis of 11 of the 16 NHs participating in MOQI; and 2) to discuss work-flow benefits of avoiding hospitalizations through early detection of changes in health status using qualitative data from the 16 NHs participating in MOQI.

## Revenue Lost to the NH Due to Hospitalizations

When NH residents are hospitalized, revenue is withheld to NHs from payer sources, particularly Medicare, Medicaid, and some private insurers, for the days each person is out of the facility. These “empty bed days” due to hospitalizations represent lost revenue for NHs that is often overlooked by NH leaders. Typically, “percentage occupancy” is the standard by which leaders judge the efficiency of their operations with most focusing efforts to achieve higher occupancy percentages. While percentage occupancy is a worthwhile indicator, these rates can also be misleading, masking revenue that cannot be billed to payers due to hospitalizations. Small amounts of non-billable days while residents are hospitalized each week can result in large amounts of revenue lost that can be recaptured by reducing hospitalizations of NH residents each year.

There is variation of policies as some states provide some reimbursement for days of hospitalization of nursing home residents, sometimes referred to as “bed hold” days.16 Twenty-one states, including Missouri where this study was conducted, do not provide “bed hold” or “empty bed days” reimbursement. Another twenty states and the District of Columbia do provide some reimbursement, but almost all limit the number of days paid and half limit the amount paid to as low as $6.28 to $10.43 per day or 30% to 75% of their Medicaid per diem rate. The remaining nine states limit payment based on occupancy, providing reimbursement only if all Medicaid beds are full or the facility has a high occupancy rate (average of 92% across these states, range 98% to 85%) (16). Clearly, non-billable days due to hospitalization of residents has a financial impact on all nursing homes across all states.

## Methods

### Sample

All 16 NHs participating in MOQI were asked to participate in this analysis. Eleven of the 16 provided necessary financial data. Participation in the analysis was voluntary and approved by the university institutional review board. Nine of the 11 NHs were urban and two were rural. The average bed size across the 11 participating NHs was 199 (range 115–321) (Table 1).

**Table 1:** Hospitalization Rates per 1000 Resident Days for Long Stay Residents 2014–2019 (n=11 NHs, bed size, urban/rural designation)

**NH/beds/urban or rural**	**2014**	**2015**	**2016**	**2017**	**2018**	**2019**
A (167) urban	1.58	2.08	1.91	1.86	1.22	1.45
B (115) rural	2.53	1.98	2.46	1.75	1.22	1.05
C (237) urban	2.87	1.96	1.46	2.98	1.03	2.48
D (132) urban	1.93	1.07	1.20	1.56	1.57	2.13
E (189) urban	1.98	2.61	1.74	2.07	1.8	1.38
F (240) urban	2.13	1.64	2.27	1.92	2.50	2.61
G (240) urban	3.81	2.82	3.01	1.89	1.75	2.00
H (180) urban	2.24	1.89	2.29	1.99	1.64	1.26
I (250) urban	2.40	2.05	1.67	2.37	2.60	2.16
J (321) urban	2.97	2.06	1.01	0.98	1.23	1.07
K (120) rural	1.49	1.54	0.62	1.43	1.22	1.08
Average (199)	2.36	1.97	1.79	1.89	1.62	1.70

### Estimating Potential Revenue Recapture (PRR) due to Hospitalization Days

Financial and occupancy data provided by each facility's administrator were used to estimate the annual amount of potential revenue recapture (PRR) (lost revenue) due to resident hospitalization days. These data are readily available from most financial systems for NH operations. Data from 2019 were readily available and provided by the NHs for analysis. Data included information about long and short stay residents so calculations of PRR (lost revenue) for non-billable days due to hospitalizations for both populations were estimated.

Specifically, long stay information from facilities included payment rates and average daily census for each payment type (Medicaid and other insurance/private pay). Similarly, short stay information from facilities included payment rates, average daily census, and average length of stay for each payment type (Medicare, Managed Medicare, and other insurance/private pay). Typically, payment rates are adjusted annually, so annual rates for 2019 were provided by each facility and used in this analysis.

The actual payment per day for each payment type was calculated using both long and short stay data. As facility-specific payment rate information informed the calculation, results are facility-specific daily PRR for non-billable days due to hospitalizations. Daily PRR was multiplied by the number of days residents were hospitalized for each payment type within the category of long or short stay. For long stay, the number of days were estimated using hospitalization rates per 1000 resident days. For short stay, the percentage of short stay residents re-hospitalized after admission to NH was used as reported on the Nursing Home Compare web site: http://www.nursinghomecompare.gov

The NHs included in this analysis were current participants in the MOQI Initiative with ongoing data collection. A key part of MOQI was to provide monthly feedback reports about hospitalizations of long stay residents to each facility individually and in aggregate (9, 17). Using the feedback reports, MOQI operations team members reinforced the methods taught to facility staff to reduce avoidable hospitalizations (9). Using the ongoing data collected in MOQI for each facility, the average annual hospitalization rate per 1000 resident days for long stay residents were calculated and used in this analysis for each of the six years 2014–2019 (Table 1). For long stay residents in participating facilities, the MOQI actual average hospitalization length of stay was seven days and used in calculations.

For short stay residents, only 2019 data from the Nursing Home Compare web site were used because the corresponding financial data from each facility was only available for 2019. Calculations required using the quality indicator “percentage of short stay residents re-hospitalized,” therefore, the short-stay portion of the calculations was held constant using 2019 data for prior years in the analysis. This approach enabled comparisons across the years that would reflect the primary focus of MOQI on long stay residents, but it also provided a more complete view of the facility-wide PRR estimate for each of the NHs that included both long and short stay residents.

### Estimating Revenue Savings to Payers due to Reduction in Hospitalizations for Long Stay Residents

Because MOQI had a primary focus on long stay residents, and accurate hospitalization rates were collected for long stay residents as part of MOQI, an estimate of revenue savings to payers due to the reductions in long stay hospitalization rates was calculated for 2014–2019. The average actual Medicare costs for a hospital stay Medicare for each year were used (18). These included $12,800 (2014), $13,200 (2015), $13,600 (2016), and $14,100 (2017); the 2017 value was used for 2018 and 2019 because those figures are not yet available (18). Numbers of hospitalizations were calculated using rates of hospitalization (Table 1) and by multiplying the number of enrollees by 365 days by hospitalization rate/1000. Then, to estimate payer revenue savings, the numbers of hospitalizations for each year were subtracted from baseline year 2014. The difference was multiplied by the average actual costs per hospital stay for Medicare (18). Similarly, to estimate the actual revenue recaptured by NHs due to reducing hospitalizations the difference was multiplied by the average long stay daily rate and the average length of stay of hospitalizations that facilities experienced during MOQI.

### Determining Work-flow Benefits

Qualitative data collection was an integral part of the MOQI Initiative. Using qualitative methods helps to elucidate contextual understanding about the conditions when, how, and why intervention methods are working. Two written questions were posited to MOQI APRNs working in their respective NHs. The APRNs were asked to “reflect on your practice in your facility the past few years” to answer the following two questions: 1) What benefits do you see in the workflow for nurses because you have taught them how to do early illness detection and avoid the need for hospitalizations? 2) For nursing assistants?

Each APRN provided written responses and submitted them electronically for analysis. An inductive content analysis was conducted using word processing software to facilitate emergence of patterns, themes, and categories19 by a qualitative researcher. A second experienced qualitative researcher reviewed the data trail and conclusions to confirm descriptive findings and increase the trustworthiness of the analysis.

## Results

### Estimated Potential Revenue Recapture (PRR) for Long and Short Stay Residents

The total PRR (lost revenue) due to non-billable days in the 11 participating NHs when residents were hospitalized during six years (2014–2019) is reported in Table 2. For each NH, for long and short stay residents who were hospitalized, PRR ranged from $590,000 to over $5 million. As a group, the 11 facilities had an additional $32.5 million in PRR due to non-billable days of both long and short stay hospitalizations during the six years. On average, there was about $500,000 in PRR (lost revenue) per year per 200 beds per NH.

**Table 2:** Estimated Potential Revenue Recapture (PRR) to NHs due to Hospitalizations of Long Stay and Short Stay Residents, 2014–2019 (n=11 NHs)

**NH**	**PRR 2014**	**PRR 2015**	**PRR 2016**	**PRR 2017**	**PRR 2018**	**PRR 2019**	**Total PRR over 6 years**
NH A	$418,462	$420,238	$387,552	$416,330	$408,869	$403,895	$2,455,348
NH B	$162,949	$180,315	$174,410	$172,674	$150,445	$158,434	$999,228
NH C	$589,894	$552,297	$585,109	$536,575	$500,345	$488,724	$3,252,945
NH D	$573,362	$536,574	$516,360	$577,809	$498,977	$557,596	$3,260,680
NH E	$586,300	$554,286	$559,125	$572,527	$572,899	$594,117	$3,439,256
NH F	$772,038	$814,967	$755,685	$778,171	$759,773	$731,154	$4,611,791
NH G	$867,074	$838,292	$875,297	$854,739	$888,807	$895,268	$5,219,480
NH H	$392,824	$351,865	$359,726	$313,388	$307,595	$317,939	$2,043,339
NH I	$806,287	$778,737	$810,222	$786,609	$759,059	$729,148	$4,670,065
NH J	$351,709	$330,171	$306,788	$349,863	$364,016	$336,940	$2,039,488
NH K	$129,623	$90,500	$89,901	$93,494	$94,892	$91,698	$590,111
TOTAL	$5,650,526	$5,448,247	$5,420,181	$5,452,181	$5,305,682	$5,304,918	$32,581,737
Average	$513,684	$495,295	$492,743	$495,653	$482,334	$482,265	$2,961,976

### Estimated Savings to Payers and Actual Revenue Recapture to NHs for Long Stay Residents

The savings to payers totaled over $31 million during the five years (2015–2019), with 2014 serving as baseline, due to reduction in long stay resident hospitalizations (Table 3). Actual recaptured revenue to NHs in this sample totaled more than $2.6 million for the same time period (i.e., 5 years) for long stay residents (Table 3).

**Table 3:** Estimated Savings to Payers and Actual Revenue Recapture to NHs due to Reduction in Hospitalization Rates for Long Stay Residents 2015-1019 Compared to Baseline 2014 (n=11 NHs)

	2014*	**2015**	**2016**	**2017**	**2018**	**2019**	**Total over 5 years**
Numbers of Hospitalizations	1076	835	751	662	457	427	4208
Estimated Savings-Payers		$3,181,200	$4,452,500	$5,837,400	$8,727,900	$9,150,900	$31,349,900
Estimated Actual Revenue Recapture-NHs		$285,103	$384,475	$489,762	$732,277	$767,767	$2,659,384


*2014 was the baseline year

### Annual Average Savings for Payers and Revenue Recapture for NHs by Reducing Hospitalizations

**Figure 1:**
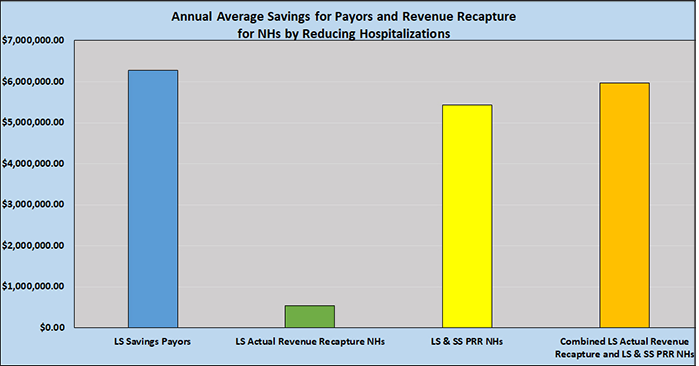
Annual Average Comparison of Long Stay Estimated Savings for Payers, Actual Long Stay Revenue Recapture NHs, Long Stay and Short Stay PRR NHs, and Combined Long Stay Actual Revenue Recapture and Long Stay & Short Stay PRR NHs Note: LS=long stay; SS=short stay; PRR=potential revenue recapture due to non-billable “empty bed” days

The first two columns of Figure 1 illustrate the key results comparing annual savings to payers and actual revenue recaptured by NHs by reducing hospitalizations for long stay residents. As shown, the payers gain much more in savings than the NHs gain in revenue by reducing “empty bed” days for long stay residents. However, when considering the PRR for both short and long stay residents for additional “empty bed” (non-billable) days due to hospitalizations, there is nearly equal additional revenue the NHs could have annually recaptured (the second and third columns that are combined in the fourth column) as savings for payers for long stay residents (the first column).

### Nurses' and Nursing Assistants' Work-flow Benefits

Fourteen of the seventeen APRNs provided written responses for the content analysis related to perceived workflow benefits to nurses and nursing assistants that were a result of improved early illness detection and reductions of potentially avoidable hospitalizations. Table 4 summarizes the categories of perceived workflow benefits for both nurses and nursing assistants.

**Table 4:** Categories of Workflow Benefits for Nurses and Nursing Assistants

**Direct Care Providers**	**Categories of Workflow Benefits**
Nurses	ability to recognize and respond to changes in condition early and treat faster
	less disruption of routine workflow saves time
	clear communication, streamlines and identifies “priority alerts”
Nursing Assistants	early recognition of change in condition limits resident decline
	early notification of change in condition occurs without interrupting workflow
	INTERACT Stop & Watch tool saves time for nursing assistants
	communication is more effective, smoother workflow, and there is a positive impact in care

### Nurses' Workflow

Three categories of benefits emerged in nurses' workflow. Each category was supported by multiple similar examples from multiple APRNs. The first category is the ability to recognize and respond to changes in condition earlier and treat faster. The APRNs reported, “Nursing assessments have improved significantly, improving the information nurses provide to clinicians when notifying them of the change,” “orders for workup and treatment are appropriate and timely, decreasing lag time to treatment and reducing hospital transfers” and “recognizing early signs and symptoms of illness and collecting information systematically; nurses are saving time and easily incorporating the management of change of conditions into their routine workflow.”

A second category in nurses' workflow was that less disruption of routine workflow saves time. For example, APRNs reported, “Change in condition orders are a lot less work than prepping to send residents out, and then readmitting with all new orders when they return,” and “reduced time with hospital transfer paperwork (updating medication administration records, calling providers to confirm orders, etc.).”

A third category in nurses' workflow was that clear communication, streamlines and identifies “priority alerts.” APRNs provided examples about specific tools that enhanced the ability to communicate effectively. For example, “Stop and Watch constitutes a “Priority Alert” for the nurse,” “using INTERACT tools [SBAR] to communicate and document changes in condition eliminates redundant charting,” and “streamlines what is to be documented and communicated not only between staff, but also to families and hospitals.” APRNs also suggest that tools not only improve communication but also guide communication, “SBARs and change in condition tools help them identify what is important and guide conversations with providers in a quick and concise manner.”

### Nursing Assistants' Work-flow

APRNs perceived improvements in nursing assistants' workflow in four categories. The first category is early recognition of change in condition limits resident decline. This category was characterized by APRNs reporting, “If Illness is detected early and managed early, resident's ADLs have less decline or recover quicker, therefore nursing assistants' workflow is steadier as well,” and “increasing overall education with disease process and prevention so they [nursing assistants] have a better idea of how to identify changes quicker. This eliminated the ongoing watch and wait process which takes time away from giving regular care.”

A second category identified is that early notification of change in condition occurs without interrupting workflow. For example, APRNs reported, “They use the [INTERACT] Stop and Watch [tool] to report early illness symptoms without interrupting their routine workflow,” and “they know that early workup and treatment is important.”

A third category identified that the INTERACT Stop and Watch tool saves time for nursing assistants. APRNs report, “Time savings when reporting change in conditions to nurses — nursing assistants quickly fill out the Stop and Watch and leave it on the desk,” “Stop and Watch provides a quick way to communicate and document changes in condition for prompt attention and intervention,” and “if Stop and Watch is not used, I find that CNA's waste time running to multiple people to communicate a change.”

Finally, the fourth category captures the importance of nursing assistant workflow on care, that communication is more effective, smoother workflow, and there is a positive impact in care. For example, “They tell someone (charge nurse) immediately about the change in condition, at the time of the event,” “we communicate more effectively and quicker—workflow better,” and “CNAs have embraced the concept of Stop & Watch and are truly excited when they see the impact their observations can make in the care of residents.”

## Discussion

There are considerable savings to payers by encouraging reductions in hospitalizations. In this sample of 11 of the 16 NHs participating in MOQI, there were more than $31 million in savings to payers during the five years (2015–2019) due to reduction in long stay hospitalizations (Table 3). For NHs, recaptured revenue due to reduction in hospitalizations totaled more than $2.6 million for those five years for long stay residents. When considering both short and long stay residents, there is considerably more potential revenue to recapture. For one facility, the PRR was about $100,000 per year due to recaptured revenue for non-billable days during hospitalizations for both short and long stay residents, while other NHs experienced seven to eight times that amount annually. As a group, PRR of total short and long stay non-billable days during hospitalizations ranged from $590,000 to over $5 million per facility for six years (2014–2019) (Table 2). Participating NHs actively focused on and successfully reduced long stay hospitalizations during the course of the MOQI Initiative (9, 14, 15, 17). As remarkably large as this PRR was, lost revenue for facilities not participating in an initiative, like MOQI that focused staff attention on reducing hospitalizations, would likely have been much larger.

Revenue is lost due to hospitalization, which results in “empty bed days” that occur on both the short and long stay portions of the NH. PRR is much higher when hospitalizations of long stay and short stay are combined, totaling $32.5 million for the 6-year analysis of the 11 NHs (Table 2). The average long stay actual revenue recapture realized by these NHs, combined with PRR, totals over $6 million annually (Figure 1). It would seem that NH leaders would be incentivized to recapture revenue by pursuing strategies such as those proven in MOQI to reduce avoidable hospitalizations. One recommendation from this analysis would be for NH leaders to weekly or monthly tabulate non-billable, “empty bed” days so they can monitor the amount of potential revenue that can be recaptured by reducing hospitalizations for long and short stay residents.

While there is state variation in payment policies for “empty bed” days, every state limits the payment in some way.16 Nearly half provide no reimbursement, others limit to very high occupancy rates, and nearly all of those who do provide some reimbursement limit the amount paid below the state per diem Medicaid rate (16). From a workload perspective, doing early illness recognition and early treatment of health changes within the facility provides for a smoother workflow, and limits the additional costs of staff focused on new admission or readmission processes.

Changing mind-sets is not easy but quite doable, as we have found in MOQI. Consistent staff education and role modeling help overcome common nursing staff reactions to resident illness and change in condition. For instance, staff reactions to a resident change in condition is often “ship to hospital”; however, more complete assessment often reveals actions that are more appropriate to manage the clinical situation and treat the resident in place at the NH (9, 20). Another recommendation is for nursing home leadership to invest in staff with the clinical skills (RNs and an APRN) to role model how to detect changes in health status early, how to do more complete assessments, communicate information to healthcare providers clearly, and guide implementing clinical interventions that help residents recover quickly within the nursing home. We have found that most diagnostic workups can be completed readily within the nursing home so that the majority of changes in health status can be managed without hospitalization (9, 20).

INTERACT is a suite of tools that include Stop & Watch and SBAR as a way to continuously improve quality surrounding resident illness identification and management. (1, 21). As outlined in the qualitative findings, these tools were perceived by the APRNs to be key to improving workflow for both nurses and nursing assistants working in MOQI facilities. Benefits of INTERACT include being able to expeditiously inform the nurse with Stop & Watch, so the nurse knows there is a “priority” assessment that needs to be done to evaluate a resident for change in condition. Nursing assistants can readily inform the nurse without major interruptions of nursing assistant workflow. SBAR provides nurses with a comprehensive strategy to assess and evaluate potential causes of condition change and consistently communicate findings and requests to providers in a concise but complete manner. This enables necessary information to be readily communicated to a health care provider with a single organized and efficient phone call that confers complete information that is more likely to result in treatment that often can be implemented within the NH, avoiding hospitalization (9, 13).

It is our recommendation that all nursing home leaders implement the use of the INTERACT suite of tools, if not all of them, then at least Stop & Watch and SBAR. Once implemented, leaders must continue to follow up and reinforce consistent use, so that staff actually use these tools every day, 24 hours each day. Qualitative findings of this study confirm the success of nurses and nursing assistants who use INTERACT tools to reduce hospitalizations. Similarly, other researchers have measured significant reductions in hospitalizations in NHs that reported increases in INTERACT use while NHs with low or moderate use had insignificant changes in hospitalizations (21, 22).

Communicating changes in condition are also enabled by health information technology. MOQI results demonstrated that in many cases, health information technology enabled communication between nursing assistants and nurses as well as nurses' communication with other health care providers without interrupting either the nurse or nursing assistants' workflow (12, 23). Communication facilitated by health information technology, INTERACT, and text message results in more timely and appropriate care for residents and increases opportunities to avoid hospitalization (23). It is our recommendation that efforts expand the use of technology in all nursing homes, connecting them to other healthcare providers, hospitals, and others.

MOQI also emphasized resident and family involvement (24) in end-of-life care and resident choice in the type of care desired at the end of life. Residents were encouraged to enact advance directives (25) and these advance directives documents were reviewed periodically to ensure that they reflected the residents wishes.25 Advance care planning and advance directives were topics for staff education in all facilities and leadership implemented facility policies on end-of-life care and advance directives. In an analysis of MOQI data, residents who had advance directives were less likely to be transferred to hospitals unnecessarily (25). It is our recommendation that nursing home leaders implement facility policies to assure all residents have clear plans for end-of-life care and advance directives and that staff routinely review and discuss these with residents and families so all are aware of resident wishes and residents can rest assured that their plans will be followed.

While hospitalizations are costly to payers (1, 2, 26) and result in non-billable days for NHs, as illustrated in this analysis and others, reducing hospitalizations also improves the quality of care (9, 27). Hospitalization accelerates functional and cognitive decline when NH residents experience the stress of relocation (6, 7). Improving early illness recognition and assessment skills of nursing staff can facilitate early diagnosis and treatment of changes in condition within the NH.

There are estimates that 67%1, 60%2 and 58% (15) of hospitalizations of NH residents are avoidable. Recapturing lost revenue from avoidable hospitalizations would more than cover the salary of an APRN to work with their nursing staff and still have additional funds available to use for other purposes. Similar to MOQI, the APRN would guide nursing staff in early illness recognition and best practices to manage common health conditions that can be promptly and safely managed within the NH. We highly recommend that all nursing homes hire their own APRN, or share one with a nearby facility, so that their staff have the advanced clinical support of an APRN and their residents have ready access to the advanced clinical skills of an APRN (28). The facility will benefit from the overall improvement in quality of care reflected not only in the care and satisfaction of families, but also in quality measures (QMs) used by state and federal regulators (27).

There are several limitations that must be considered in generalizing results. This analysis is limited to a small sample size, eleven of 16 facilities participating in MOQI. All 16 facilities were asked to participate, 5 did not provide the necessary financial data for analysis; it is unknown why they chose not to participate or how their participation would have influenced the findings. The facilities are in one state, and generally one region of that state, including both urban, suburban, and rural locations. One strength of the analysis is that it is longitudinal over six years. The extended duration allows for examining consistency of the results over time.

## Conclusions

Reducing avoidable hospitalizations has large benefits for reducing costs to payers, but there are also substantial financial benefits to NHs as they recapture revenue that is lost with each day of hospitalization of their residents. Focusing attention of nursing staff on early illness recognition and expeditious management of change in condition within NHs has benefits for residents, too. Residents can avoid the additional stress of hospital transfer and functional decline associated with it. Nurses and nursing assistants can benefit from improvements in their workflow by focusing on early illness detection, managing most changes in health conditions within the NH, and reducing avoidable hospitalizations. Nursing homes need to engage the services of an APRN to benefit their overall quality of care and provide early illness recognition and skilled follow up care to residents.
